# Gut Microbiota–Bile Acid–Brain Axis and TGR5‐ERK1/2 Signaling Mediate ADT‐Induced Cognitive Impairment

**DOI:** 10.1111/cns.70608

**Published:** 2025-09-15

**Authors:** Fan Yang, Yanbo Liu, Zhien Zhou, Dong Yang, Weigang Yan

**Affiliations:** ^1^ Department of Urology, Peking Union Medical College Hospital Chinese Academy of Medical Sciences and Peking Union Medical College Beijing China; ^2^ Department of Anesthesiology, Plastic Surgery Hospital Chinese Academy of Medical Sciences and Peking Union Medical College Beijing China

**Keywords:** androgen deprivation therapy, bile acid metabolic reprogramming, cognitive dysfunction, gut microbiota, prostate cancer, taurodeoxycholic acid, TGR5

## Abstract

**Aims:**

Although a key intervention for advanced Prostate Cancer, Androgen Deprivation Therapy has been associated with cognitive dysfunction, a phenomenon that has been largely attributed to systemic metabolic alterations and neuroinflammation. Nonetheless, the precise role of gut microbiota in ADT‐induced cognitive impairment remains unclear, forming the basis of this study. Our aim is to explore the correlation between changes in gut metabolism and cognitive dysfunction following ADT in prostate cancer.

**Methods:**

A subcutaneous PC3 tumor‐bearing mouse model of ADT‐induced cognitive dysfunction was established. Behavioral tests (OFT, NORT, and Y‐maze) were conducted to assess cognitive performance. Gut microbiota composition, fecal, and hippocampal bile acid profiles were analyzed by 16S rRNA sequencing and targeted metabolomics. To investigate potential mechanisms, we further supplemented ADT‐susceptible (ADT‐su) mice with Taurodeoxycholic acid (TDCA) via oral gavage and inhibited ERK1/2 signaling with PD98059, followed by behavioral testing and Western blot analysis of hippocampal Takeda G‐protein coupled Receptor 5 (TGR5) and ERK1/2 expression.

**Results:**

Hierarchical clustering analysis revealed ADT‐induced cognitive impairment in a subset of mice (ADT‐susceptible and ADT‐unsusceptible). These mice exhibited gut microbiota dysbiosis, featuring the depletion of bile acid‐transforming taxa, including *Bacteroides* spp. and *Clostridium scindens* . Additionally, FMT from ADT‐su mice to pseudo‐germ‐free mice efficiently transferred cognitive deficits and altered hippocampal bile acid profiles, confirming gut microbiota's causal role in ADT‐induced neurocognitive decline. Notably, both gut and hippocampal TDCA levels were significantly decreased in ADT‐su mice. Mechanistically, TDCA supplementation improved cognitive performance and upregulated hippocampal TGR5 and p‐ERK1/2 expression, while ERK1/2 inhibition by PD98059 partially reversed these effects.

**Conclusion:**

Our findings suggest that gut microbiota‐mediated bile acid dysregulation, particularly reduced TDCA, contributes to ADT‐induced cognitive dysfunction via impaired TGR5‐ERK1/2 signaling. Targeting this pathway may represent a novel therapeutic strategy to mitigate cognitive impairment in prostate cancer patients undergoing ADT.

## Graphical abstract

Our study shows how androgen deprivation therapy (ADT) affects prostate cancer patients, leading to impaired cognition. It shows the impact of ADT on gut microbiota dysbiosis, bile acid metabolism, and the blood–brain barrier, with changes in Taurodeoxycholic Acid and specific microbial species like Acinetobacter sp. and *Acinetobacter calcoaceticus* . Reduced hippocampal TDCA leads to diminished activation of TGR5 and the ERK1/2 signaling pathway, promoting neuroinflammation and oxidative stress and ultimately contributing to cognitive dysfunction. Supplementation with TDCA restores TGR5/ERK1/2 activation and confers neuroprotection.

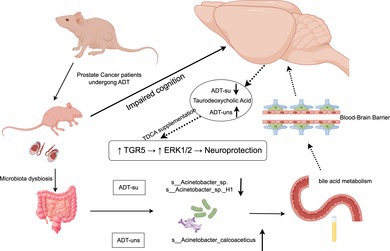

## 1 Introduction

Androgen Deprivation Therapy (ADT), a standard intervention for advanced Prostate Cancer (PCa), could effectively reduce androgen levels or block androgen receptor signaling, effectively suppressing tumor growth and progression. Consequently, ADT could significantly improve survival outcomes in patients with metastatic and advanced locoregional diseases [1, 2]. ADT aims to suppress the production of androgens or inhibit androgen receptor signaling, thereby limiting the growth and survival of androgen‐dependent prostate cancer cells. Clinically, ADT can be achieved through surgical castration (bilateral orchiectomy) or medical castration using gonadotropin‐releasing hormone (GnRH) agonists, GnRH antagonists, and androgen receptor pathway inhibitors such as enzalutamide and abiraterone. Despite its efficacy in controlling tumor progression, long‐term ADT is associated with multiple adverse effects, including metabolic disturbances, cardiovascular risks, osteoporosis, and neurocognitive impairment, significantly impacting the patients' quality of life [3].

Long‐term ADT exposure is linked to neurocognitive deficits—such as impaired memory, executive function, and spatial processing—though the underlying mechanisms remain unclear despite their major impact on patients' quality of life and treatment adherence [4, 5, 6]. Preclinical studies suggest testosterone deprivation may impair hippocampal neurogenesis and synaptic plasticity through BDNF downregulation and amyloid‐β accumulation [7]. Systemic inflammation may also contribute, as ADT‐induced hypogonadism can raise IL‐6 and TNF‐α levels, disrupt the blood–brain barrier, and worsen neurotoxicity [8].

The gut–brain axis is a critical pathway linking systemic physiological changes to brain function [9]. The gut microbiota, a complex community of microorganisms localized in the gastrointestinal (GI) tract, is crucially involved in maintaining health and regulating various metabolic and immune processes. In this regard, it is noteworthy that dysbiosis, an imbalance in gut microbiota composition, has been implicated in various neurocognitive disorders, including Alzheimer's Disease (AD) and chemotherapy‐induced cognitive impairment (CICI) [10]. Furthermore, gut microbiota‐produced metabolites, such as short‐chain fatty acids (SCFAs), neurotransmitter precursors, and inflammatory mediators could influence brain metabolism and function [11].

The hippocampus, a key brain region that regulates learning and memory, is particularly susceptible to metabolic disruptions [12]. Systemic and local change‐induced metabolic reprogramming within the hippocampus could alter neuronal activity, synaptic plasticity, and neurogenesis, causing cognitive decline. Moreover, the interplay between neuroinflammation and metabolic changes could further complicate these processes. It is also noteworthy that chronic neuroinflammation could impact adult‐born neuron recruitment into hippocampal networks, affecting cognitive functions [13]. In the context of ADT, the potential interplay between altered gut microbiota, its metabolites, and hippocampal metabolic reprogramming remains an unexplored yet highly promising area of research.

Herein, we aimed to explore the role of gut microbiota and its metabolites in hippocampal metabolic reprogramming associated with ADT‐induced cognitive impairment in PCa mouse models. We mapped an atlas of the gut–brain axis in mice with ADT‐induced cognitive dysfunction using 16S rRNA sequencing, untargeted metabolomics, and behavioral tests. Besides illuminating the mechanisms underlying the relationship between ADT and cognitive dysfunction, our findings highlight potential therapeutic targets for ADT‐induced neuro‐degradation in PCa survivors.

## 2 Materials and Methods

### 2.1 Animals

Male Balb/c Nude mice (age = 6–8 weeks) were acquired from the Beijing Vital River Laboratory Animal Technology Co. Ltd., (Beijing, China) and kept in standard housing conditions [room temperature (RT) = 20°C–26°C; relative humidity (RH) = 40%–70%; and 12/12 h light/dark cycles], awaiting tests. Human PCa PC3 cells were cultured in RPMI‐1640 medium supplemented with 10% Fetal Bovine Serum (FBS) and 1% penicillin–streptomycin. At 70% to 80% confluence, the cells were detached using 0.25% trypsin‐EDTA, washed with PBS, and then resuspended in serum‐free RPMI‐1640. Subsequently, the cell suspension was quantified and adjusted to a final concentration of 1 × 10^7^ cells/mL before mixing with Matrigel at a 1:1 ratio to enhance tumor formation in vivo. Each animal was injected subcutaneously into the right flank with PC3 cells (5 × 10^6^ cells in 100 μL suspension) using a 27G needle. Following that, the mice were monitored daily for tumor growth, general health status, and body weight changes. Tumor size was measured every 3 days using calipers, and the volume was calculated as follows: Tumor volume = 1/2 (length × width^2^). This study was approved by Peking Union Medical College Hospital's Animal Care and Use Committee (Approval no: XHDW‐2024‐193).

### 2.2 ADT via Castration

First, the animals were randomly categorized into two groups: Control (sham surgery, 6 mice) and ADT (castration, 12 mice). The ADT group underwent surgical castration under general anesthesia with pentobarbital sodium solution (50 mg/kg, i.p). The testes were then removed via a small scrotal incision before suturing the wound with a sterile surgical thread. On the other hand, the control group underwent sham surgery, with the testes exposed but not removed. Postoperative care encompassed analgesia administration and monitoring for signs of distress or infection. The animals were allowed 17 days to recover before performing subsequent experiments [14].

### 2.3 Behavioral Test

Both the ADT and control groups underwent behavioral experiments 17 days post‐surgery. The Open Field Test (OFT) was used to examine general locomotion, anxiety, and repetitive behaviors, while the Y‐maze test was used to evaluate spatial working and reference memory. On the other hand, the Novel Object Recognition (NOR) test was used to assess nonspatial visual learning and memory. In all tests, the experimental apparatus was cleaned with medicinal alcohol after each session to minimize odor. Behavioral data were automatically captured and analyzed using intelligent video tracking software.

### 2.4 Open Field Test

After a 3‐h habituation period, the animals were placed individually at the center of an opaque open‐field chamber (40 cm × 40 cm × 40 cm). They were allowed free movement for 5 min before performing the test, which was conducted under dim light conditions (300 lx). Total travel distance and time spent at the center area of the chamber were recorded and analyzed using an automated video tracking system.

### 2.5 NOR Test (NORT)

The NORT test was conducted as described in previous research [15, 16]. First, two identical objects were placed on the opposite sides of the arena, each positioned 6 cm from the nearest wall. During training, the animals were allowed to explore the empty arena for 5 min after habituation. After 2 h, the animals were reintroduced into the arena for a 5‐min test session, during which one of the identical objects was replaced with a novel object. Time spent exploring the novel object (NT) and familiar object (FT) was recorded. The Recognition Index (RI) was calculated as follows: RI = NT/(NT + FT).

### 2.6 Y‐Maze

The Y‐maze test was conducted in a Y‐shaped apparatus with three arms positioned at 120° angles [17]. Each arm was 30 cm in length, 8 cm in width, and 15 cm in height. During training, two arms (the start and familiar arms) were left open, while the third arm (the novel arm) was blocked. The animals were placed in the start arm and allowed to freely explore the two open arms for 5 min. After a 2‐h retention interval, all three arms were opened, and the animals were allowed to explore the maze for an additional 5 min. Time spent in each arm and the number of entries into the novel arm were recorded. The proportion of time spent in the novel arm was calculated to assess spatial working memory. Behavioral data were captured and analyzed using automated video tracking software.

### 2.7 Clustering Analysis of Cognitive Function in ADT Mice

To identify subgroups of ADT‐treated mice with differential cognitive responses, we performed an unsupervised clustering analysis based on outcomes from two behavioral tests: time spent in the new arm of the Y‐maze test and the Recognition index of NORT. Behavioral data were first standardized using *Z*‐score normalization. Hierarchical clustering was then conducted using Ward's linkage method with Euclidean distance as the dissimilarity metric. The resulting dendrogram revealed two distinct clusters, which we defined as ADT‐susceptible (ADT‐su, exhibiting pronounced cognitive impairment) and ADT‐unsusceptible (ADT‐uns, showing mild or no cognitive deficits). These stratified subgroups were subsequently used for downstream gut microbiota and metabolomic profiling to investigate underlying mechanistic differences.

### 2.8 Pseudo Germ‐Free Mice and Fecal Microbial Transplantation

Gut microbiota depletion and fecal microbial transplantation (FMT) were conducted per previously established protocols. To eliminate gut bacteria, the animals received a daily oral gavage of an antibiotic cocktail (ABX) [vancomycin (100 mg/kg), neomycin sulfate (200 mg/kg), metronidazole (200 mg/kg), and ampicillin (200 mg/kg)] for four consecutive days, significantly reducing the intestinal microbiota, thus generating a pseudo‐germ‐free mouse model. Fecal samples were collected from all mice at the end of the intervention period prior to posttreatment testing. Genomic DNA was extracted from approximately 60 mg of fecal material using the DNeasy PowerSoil Pro Kit (Qiagen, Germantown, Maryland) following the manufacturer's protocol, yielding a final volume of 50 μL DNA solution. DNA concentrations were measured using 1 μL of the extracted DNA with a Qubit fluorometer (Thermo Fisher Scientific) employing the high‐sensitivity dsDNA assay kit [18, 19].

For FMT, fecal samples were first collected separately from donor mice in the ADT and control groups. The feces were homogenized in Phosphate‐Buffered Saline (PBS) to a final concentration of 0.125 g/mL. Each mouse received 0.15 mL fecal suspension via oral gavage once daily for 3 days.

The pseudo germ‐free mice were divided into three groups based on donor microbiota: F‐ADT‐CD (ADT‐cognitive dysfunction), F‐ADT‐NCD (ADT‐non‐cognitive dysfunction) and F‐Control. Behavioral tests were conducted on the day after the final FMT session. Figure 4A details the experimental setup and timeline.

### 2.9 16S rRNA Microbiome Sequencing

Fecal samples were collected immediately after behavioral experiments, promptly frozen, and stored at −80°C, per established protocols. The LC‐Bio Technology Co. Ltd. (Hangzhou, China) performed the 16S rRNA sequencing of gut microbiota. Library preparation, DNA extraction, PCR amplification, and sequencing were all performed on an Illumina platform (San Diego, CA, USA). Raw sequencing data in FASTQ format were generated and processed. The raw data were then subjected to further bioinformatic analysis, including quality control, Operational Taxonomic Unit (OTU) clustering, and taxonomic classification using software provided by LC‐Bio Technology Co. Ltd.

### 2.10 GC–MS/LC–MS Analysis of Fecal Metabolites and Bile Acid Metabolomics in the Hippocampus

The GC–MS/LC–MS (Gas Chromatography–Mass Spectrometry/Liquid Chromatography‐Mass Spectrometry) analysis steps included sample pretreatment (fecal or hippocampal tissue), metabolite extraction, Sex Hormone Analysis (SHA), full‐scan LC–MS detection, data processing, and statistical analysis. The experiments were performed using a Dionex U3000 UHPLC system coupled with a QE Plus High‐Resolution Mass Spectrometer (HR‐MS). Raw LC–MS data were processed using Progenesis QI V2.3 software (Nonlinear Dynamics, Newcastle, UK). The contribution of each variable to group differentiation was assessed using Variable Importance in Projection (VIP) scores from the Orthogonal Partial Least Squares Discriminant Analysis (OPLS‐DA) model. The statistical significance of metabolite differences between groups was determined using a two‐tailed Student's *t*‐test. The criteria for identifying differential metabolites were VIP scores > 1.0 and *p*‐values < 0.05.

### 2.11 Validation of the Underlying Mechanism Following Bile Acid Supplementation

To further investigate the role of the TGR5‐ERK1/2 signaling pathway in ADT‐induced cognitive dysfunction, we established an extended experimental design comprising four groups of ADT‐treated mice: ADT‐uns, ADT‐su, ADT‐su with oral gavage of Taurocholic Acid (TDCA, HY‐B1899, MCE), and ADT‐su with oral gavage of TDCA combined with PD98059, a specific ERK1/2 inhibitor.

ADT‐su mice received TDCA supplementation via oral gavage once daily for seven consecutive days at a dose of 120 mg/kg/day [20], prepared in sterile PBS. In the combined treatment group, PD98059 was administered intraperitoneally (i.p.) at 5 mg/kg 30 min prior to each TDCA gavage.

Following the treatment course, all groups were subjected to behavioral testing, including the Open Field Test (OFT), Novel Object Recognition Test (NORT), and Y‐maze, as previously described. After completion of behavioral experiments, mice were euthanized, and hippocampal tissues were harvested and immediately frozen in liquid nitrogen for subsequent Western blot (WB) analysis.

For WB, hippocampal tissues were homogenized and lysed, and protein concentrations were quantified using a BCA assay. Equal amounts of protein were separated by SDS‐PAGE, transferred to PVDF membranes, and probed with the following primary antibodies: anti‐ERK1/2 (Abcam, ab184699), anti‐phospho‐ERK1/2 (p‐ERK1/2; Abclonal, AP0234), anti‐β‐Actin (Abclonal, AC038), and anti‐TGR5 (Novus, NBP2‐23669). Secondary antibodies conjugated to HRP were used, and signals were detected by enhanced chemiluminescence (ECL).

Band intensities were quantified using ImageJ software, and relative expression levels of p‐ERK1/2 and TGR5 were normalized to total ERK1/2 and β‐Actin, respectively.

### 2.12 Evans Blue Permeability Assay

A 2% Evans blue solution (4 mL/kg, Sigma Aldrich) prepared in sterile saline was administered via intravenous injection and allowed to circulate in the mice for 2 h prior to euthanasia. Following transcardial perfusion with PBS, the brains were carefully removed and weighed. Tissue samples were then homogenized in n,n‐dimethylformamide (Sigma Aldrich) and incubated at 60°C for 72 h. After centrifugation, the absorbance of the resulting supernatant was determined using a spectrophotometer (Molecular Devices, Sunnyvale, CA) at 620 nm.

### 2.13 Enzyme‐Linked Immunosorbent Assay (ELISA)

Serum concentrations of IL‐6 and TNF‐α in mice were measured using the respective ELISA kits (Boster Co. Ltd.) according to the manufacturer's instructions.

### 2.14 Statistical Analysis

All quantitative data were presented as mean ± standard error of the mean (SEM), with error bars indicating SEM. Normality was assessed using the Kolmogorov–Smirnov test, and group differences were analyzed using one‐way Analysis of Variance (ANOVA), followed by Tukey's multiple comparisons test. Results with *p* < 0.05 were considered statistically significant. All statistical analyses and graph generations (excluding specific data from 16S rRNA microbiome sequencing and LC–MS) were performed using GraphPad Prism 6.0 and Adobe Photoshop 22.1.1.

## 3 Results

### 3.1 ADT‐Induced Cognitive Dysfunction and Dysbiosis in Mice

**FIGURE 1:**
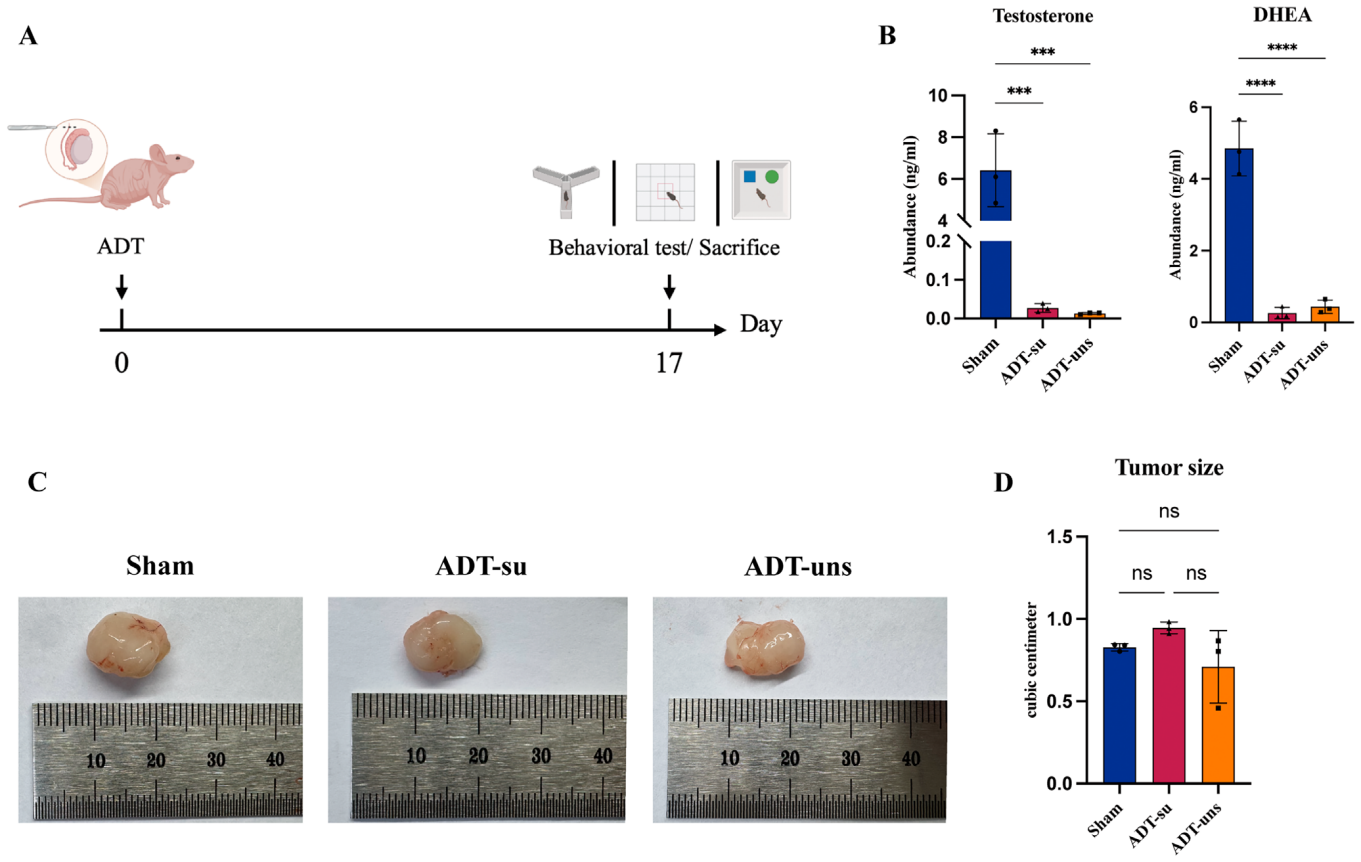
ADT establishment in PC3 tumor‐bearing mice. (A) The ADT protocol; (B) Serum androgen levels across the three groups; (C, D) No significant changes were observed in tumor volume (Tumor volumes were individually measured for each of the mice in each group). Data are presented as mean ± SEM. Group differences were analyzed using one‐way ANOVA followed by Tukey's multiple comparisons test. ****p < 0.0001, ***p < 0.001; ns, not significant.

The PC3 tumor‐bearing mouse model used in this study was constructed using Balb/c nude mice. All PC3 tumor‐bearing mice underwent ADT as outlined in Figure 1A. After 17 days, the mice underwent several behavioral tests, including the Y‐maze, OFT, and NORT. The animals were then euthanized for sample collection and subsequent analyses. Serum testosterone and dihydrotestosterone levels were measured using LC/MS, revealing significantly lower androgen levels in the ADT group than in the Sham group, thus confirming the successful establishment of the ADT model (Figure 1B). There were no significant changes in tumor volume (Figure 1C,D).

**FIGURE 2:**
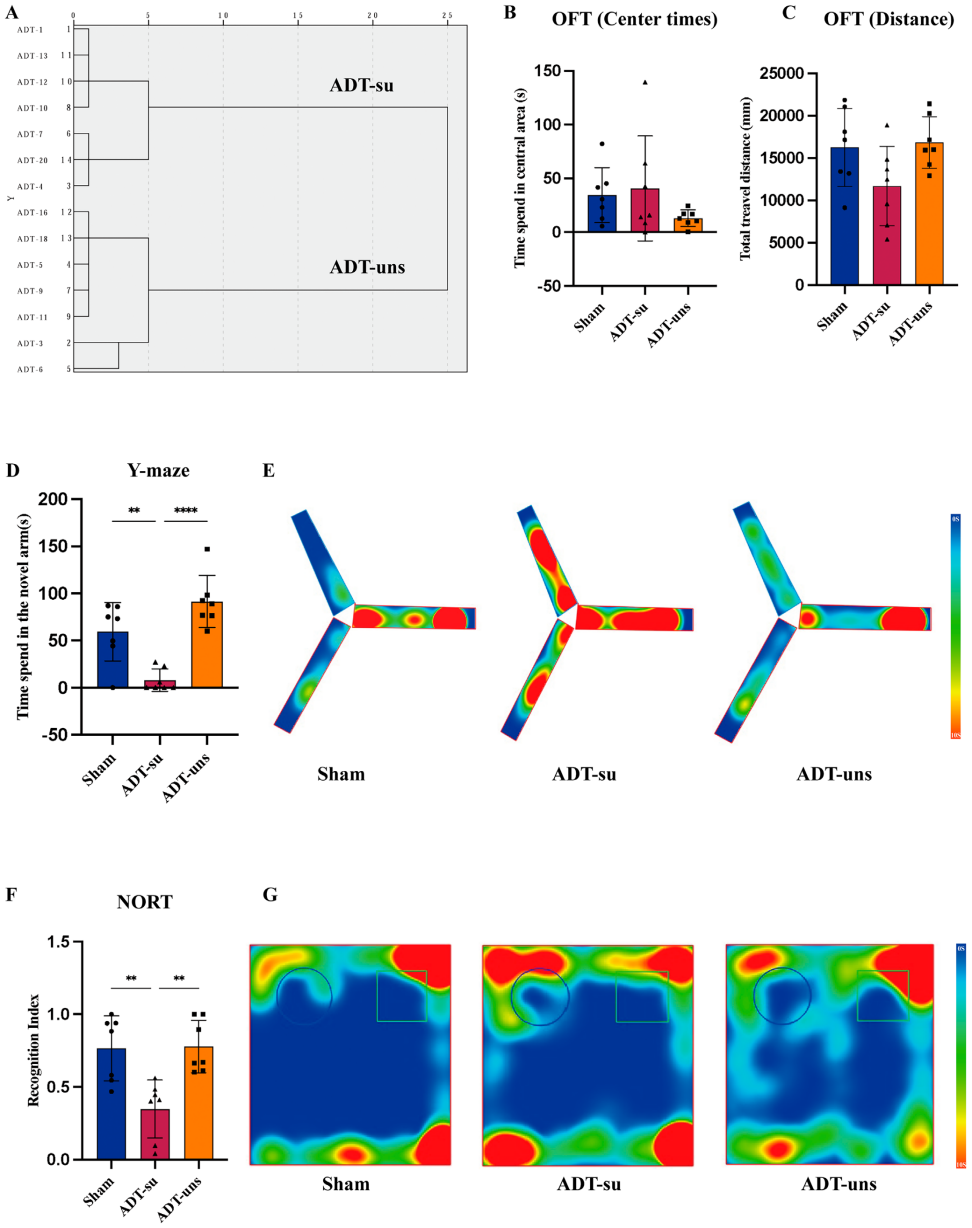
ADT‐induced cognitive dysfunction mice subjected to hierarchical clustering analysis and behavioral tests: (A) Dendrogram of the hierarchical clustering analysis; (B) Time spent in the central area of the OFT; (C) Total distance traveled in the OFT; (D) Time spent in the novel arm of the Y‐maze; (E) Representative heatmaps of the Y‐maze; (F) Recognition index of the NORT; and (G) Representative heatmaps of the NORT. Data are presented as mean ± SEM. Group differences were assessed using one‐way ANOVA with Tukey's post hoc test. **p < 0.01; ****p < 0.0001; ns, not significant.

To further establish the ADT‐cognitive function relationship, the animals were subjected to hierarchical clustering analysis based on time spent in the novel arm of the Y‐maze test and the RI in the NORT (Figure 2A). In the OFT, the three groups showed no significant differences in time spent in the central zone (Figure 2B) or total distance traveled (Figure 2C). In the Y‐maze test, we observed that the ADT‐su group spent significantly less time in the novel arm compared to both the control and ADT‐uns groups (Figure 2D), as demonstrated by representative heatmaps (Figure 2E). Moreover, compared to the other two groups, the ADT‐su group exhibited a significantly lower RI in the NORT (Figure 2F), as demonstrated by representative heatmaps (Figure 2G). These findings collectively suggest that the ADT‐su group experienced memory and learning impairments.

**FIGURE 3:**
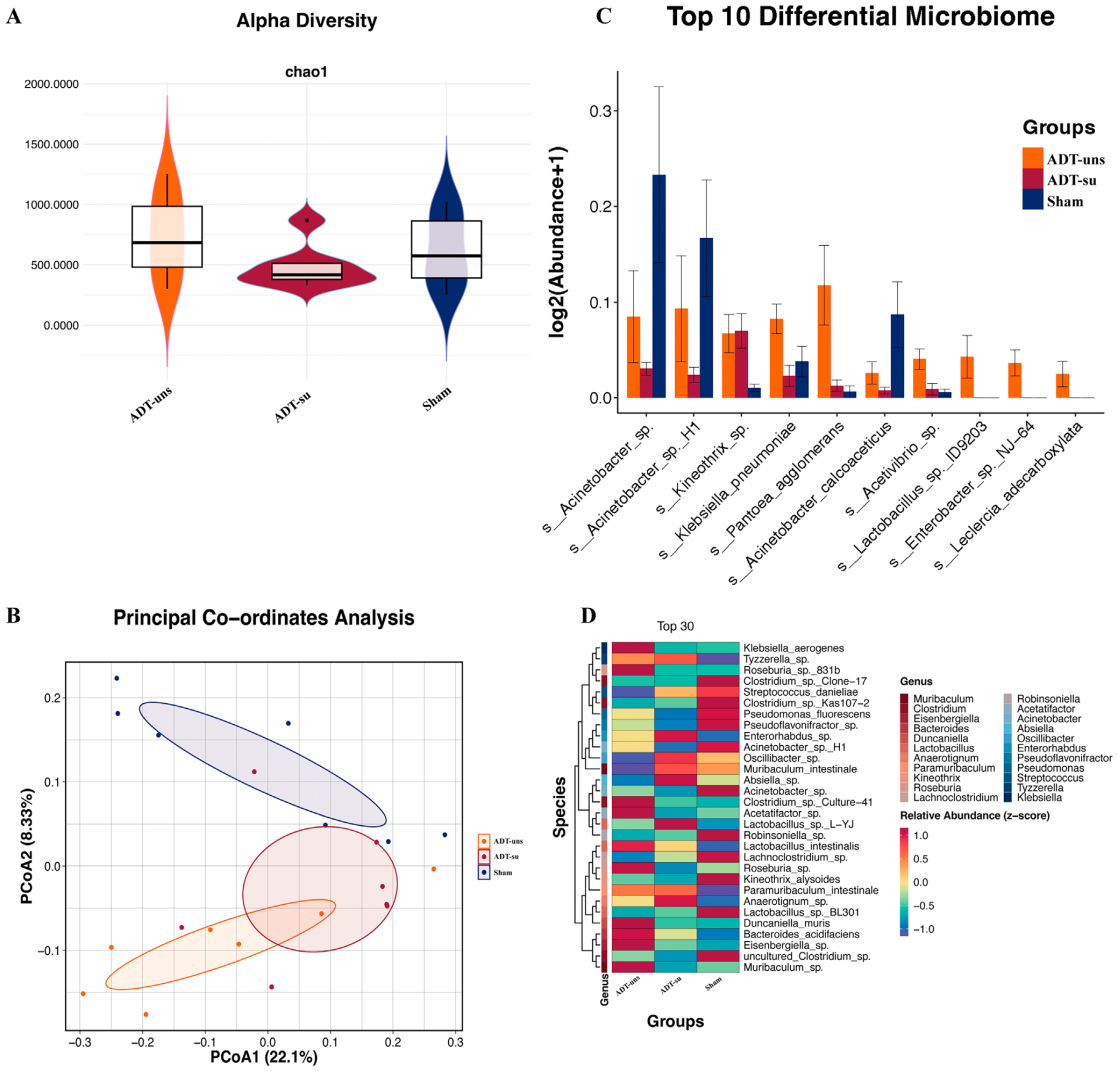
(A) Alpha diversity (Chao1 index); (B) Top 10 differential microbiomes; (C) Beta diversity (PCoA plot); and (D) Top 30 differentially abundant species at the species level. Data are presented as mean ± SEM. Alpha diversity (Chao1 index) was analyzed using the Kruskal–Wallis test. Beta diversity (PCoA) differences were evaluated using PERMANOVA. Differential taxa were identified using LEfSe analysis with an LDA threshold of > 2.0.

Fecal samples were collected from the three groups and then subjected to 16S rRNA sequencing to further explore the potential association between ADT‐induced cognitive impairment and gut microbiota dysbiosis. The ADT‐su group exhibited gut microbiota dysbiosis. Meanwhile, the three groups showed no significant differences in α‐diversity (Figure 3A), which was subjected to Principal Coordinate Analysis (PCoA) (Figure 3B), revealing considerable intergroup differences in gut microbiota composition. Figure 3C shows the top 10 differentially abundant bacterial species among the groups. In the ADT‐su group, *s__Acinetobacter_sp*. and *s__Acinetobacter_sp._H1* were significantly downregulated. On the other hand, *s__Acinetobacter_calcoaceticus* was only significantly increased in the ADT‐uns group, while the significant elevation of *s__Kineothrix_sp*. was exclusive to the ADT‐su group. Figure 3D shows the top 30 differentially abundant species at the species level, along with their corresponding gene‐level classifications.

### 3.2 FMT‐Induced Cognitive Deficits in Pseudo Germ‐Free Mice

**FIGURE 4:**
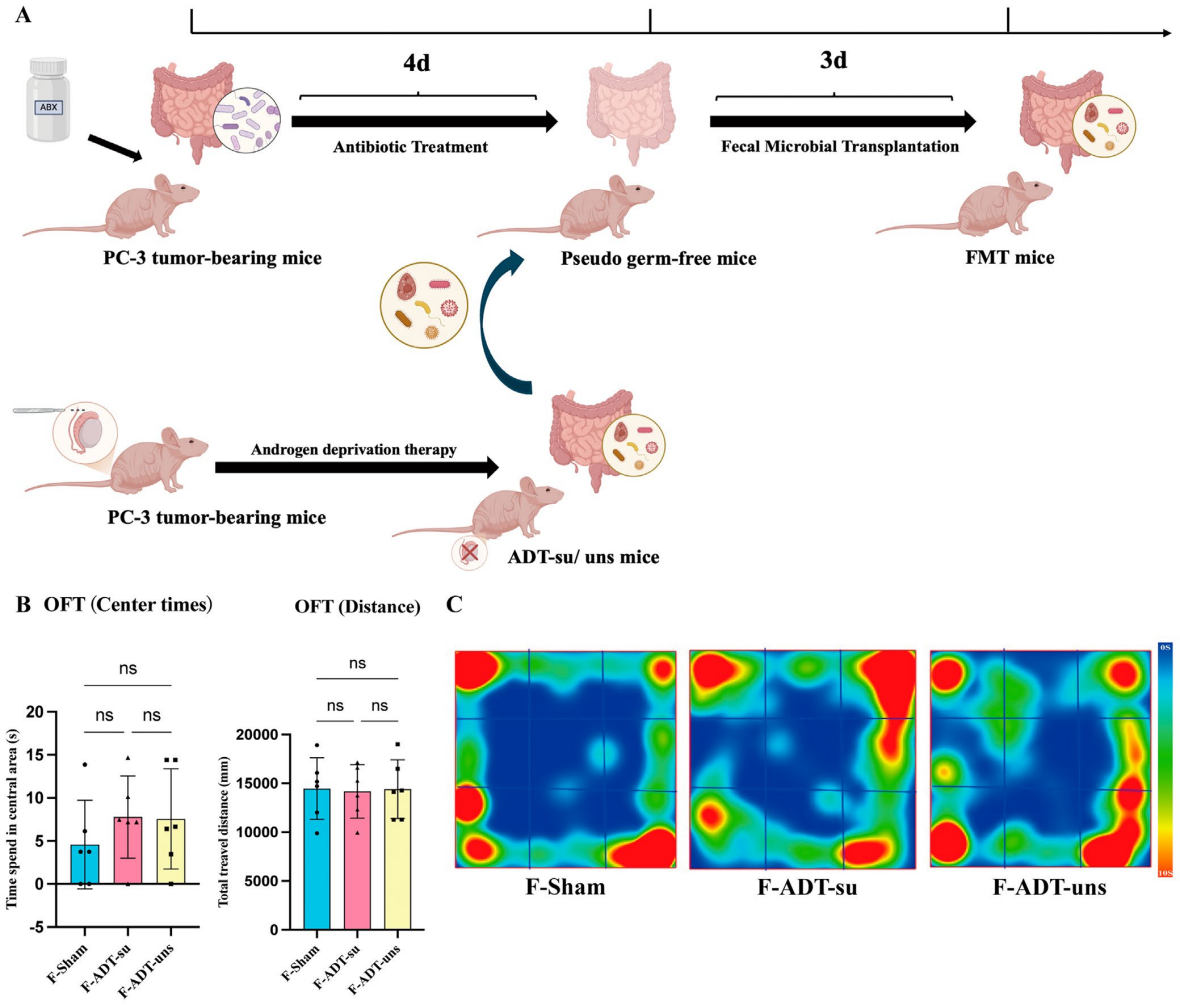
(A) Pseudo germ‐free mice preparation; (B) Total distance traveled and time spent in the central area in the OFT; and (C) Representative heatmaps of the OFT. Data are presented as mean ± SEM. Group differences in Open Field Test parameters were assessed by one‐way ANOVA followed by Tukey's multiple comparisons test.

Figure 4A illustrates the process of preparing pseudo germ‐free mice. The concentration of fecal microbial DNA differed markedly between the control and ABX groups. DNA was extracted from fecal samples of 6 control mice and 18 ABX mice at the end of the intervention. The control group exhibited significantly higher DNA concentrations, with a mean value of [51.83 ± 11.13] ng/μL, compared to the ABX group, which showed markedly reduced levels of [5.167 ± 2.431] ng/μL. Statistical analysis using the Mann–Whitney U test confirmed this difference was highly significant (*p* < 0.001), indicating effective depletion of the gut microbiota following antibiotic treatment (Figure S1A). After preparation, the pseudo‐germ‐free mice were randomly divided into three groups and subjected to FMT using samples from donor mice in the Sham, ADT‐su, and ADT‐uns groups. After microbiota transplantation, the three groups underwent OFT, NORT, and Y‐maze tests, revealing no significant differences in total distance traveled or time spent in the central area in the OFT. This finding was consistent with the OFT performance of their corresponding microbiota donor mice (Figure 4B,C).

**FIGURE 5:**
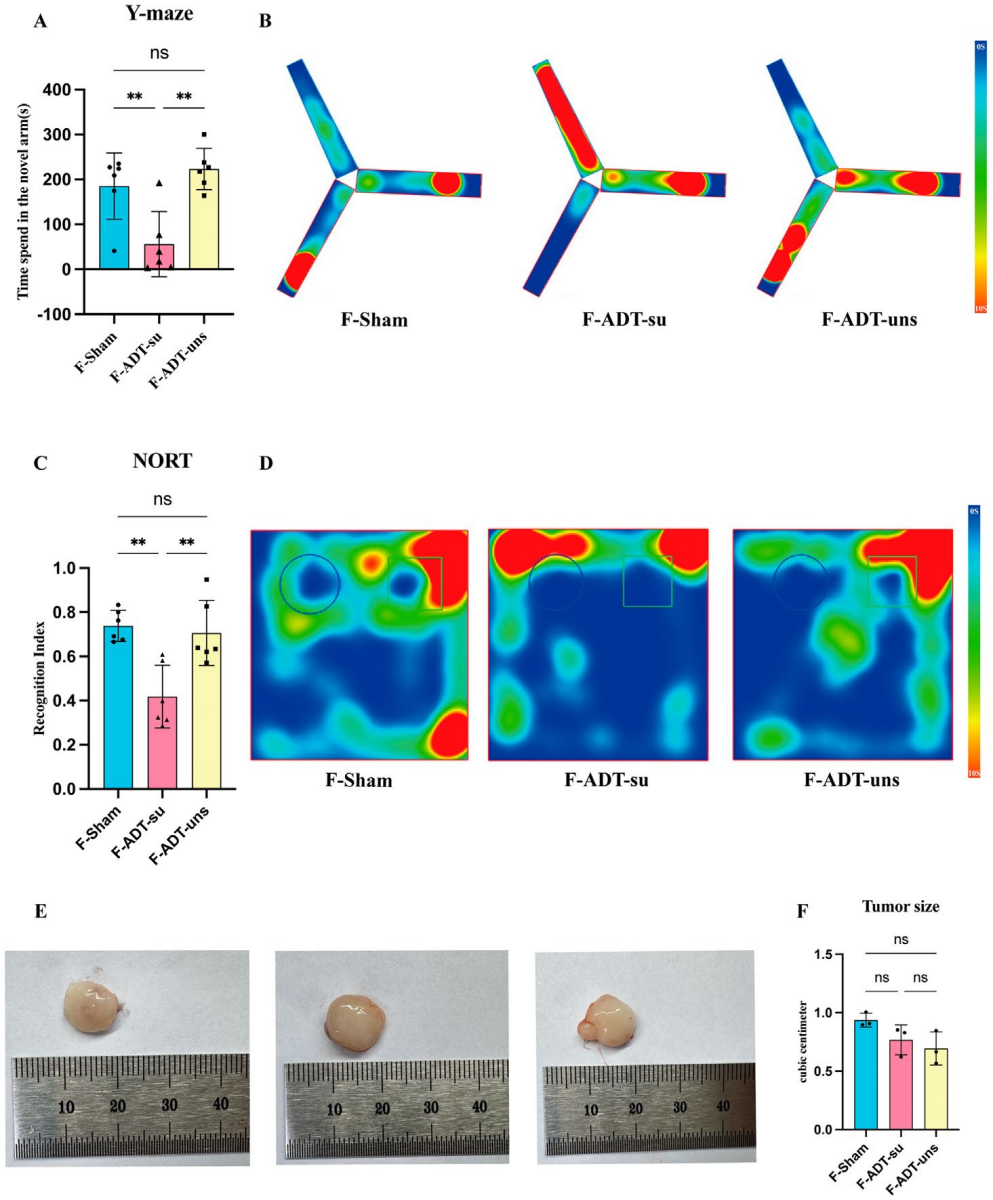
(A) Time spent in the novel arm of the Y‐maze; (B) Representative heatmaps of the Y‐maze; (C) Recognition index of the NORT; (D) Representative heatmaps of the NORT; (E, F) No significant changes were observed in tumor volume. Data are presented as mean ± SEM. **p < 0.01; ns, not significant. Data are presented as mean ± SEM. Statistical analysis was performed using one‐way ANOVA with Tukey's multiple comparisons test. **p < 0.01; ns, not significant.

Interestingly, in the Y‐maze test, FMT mice that received gut microbiota from ADT‐su group donor mice spent significantly less time exploring the novel arm (Figure 5A), as demonstrated by representative heatmaps (Figure 5B). Compared to the other two groups, these mice also exhibited a significantly lower RI in the NORT (Figure 5C), as demonstrated by representative heatmaps (Figure 5D). These behavioral findings suggest that gut microbiota dysbiosis could induce cognitive impairment in ADT‐su mice, primarily in the form of learning and memory impairment. There were no significant changes in tumor volume (Figure 5E,F).

### 3.3 Differences in Metabolites of Gut Flora in ADT‐Induced Cognitive Dysfunction Mice

**FIGURE 6:**
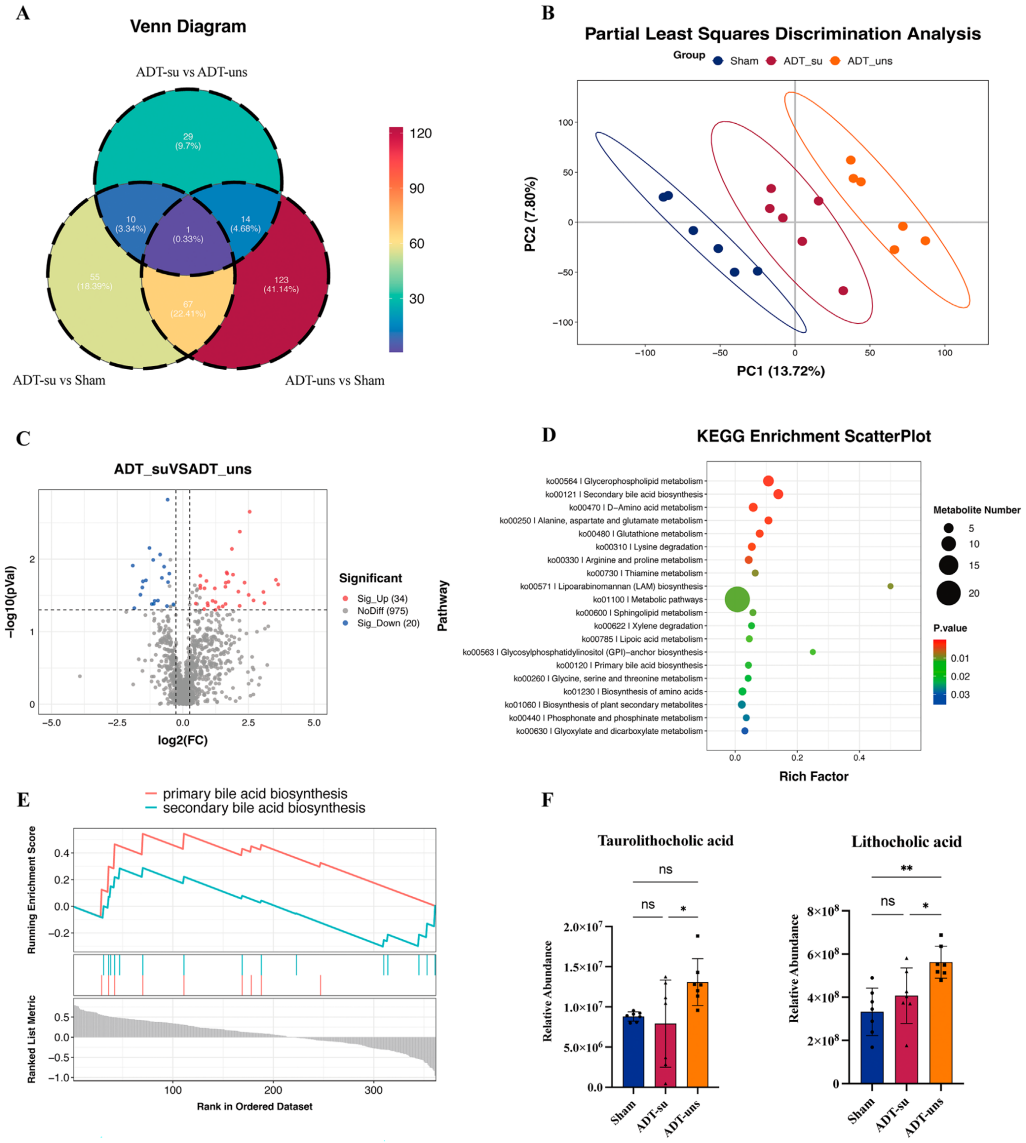
Untargeted metabolomics analysis of fecal metabolites. (A) A Venn diagram of differential metabolites across the groups; (B) PLS‐DA plot; (C) Volcano plot of differential metabolites between the ADT‐su and ADT‐uns groups; (D) Bubble plot of KEGG enrichment analysis; (E) GSEA plot; and (F) Representative heatmaps of bile acids showing significant differences across the groups. Data are presented as mean ± SEM. Multivariate analysis was performed by Partial Least Squares Discriminant Analysis (PLS‐DA). Univariate group comparisons were conducted using Student's *t*‐test. KEGG enrichment was based on Fisher's exact test. *p < 0.05; **p < 0.01.

Fecal samples were collected from Sham, ADT‐su, and ADT‐uns mice and then subjected to untargeted metabolomic analysis to further explore the relationship between gut microbiota dysbiosis and ADT‐induced cognitive dysfunction. A Venn diagram depicting differential metabolites across the groups was generated, revealing that the ADT‐su group had 54 and 133 differential metabolites compared to the ADT‐uns and Sham groups, respectively. On the other hand, the ADT‐uns group had 205 differential metabolites compared to the Sham group (Figure 6A). Partial least squares discriminant analysis (PLS‐DA) illustrated intergroup distribution, revealing substantial differences in gut microbiota metabolites across the three groups (Figure 6B). A volcano plot was also generated to visualize the differential metabolites between the ADT‐su and ADT‐uns groups, with the former exhibiting 34 and 20 significantly upregulated and downregulated metabolites, respectively (Figure 6C).

The Kyoto encyclopedia of genes and genomes (KEGG) enrichment analysis was performed (Figure 6D), revealing a considerable number of differential metabolites enriched in the primary and secondary bile acid biosynthesis pathways. Host bile acid metabolism has been closely associated with gut microbiota, and bile acid metabolism disorders could lead to cognitive impairment. Consequently, the primary and secondary bile acid biosynthesis pathways were further subjected to Gene Set Enrichment Analysis (GSEA) (Figure 6E). According to the results, gut microbiota dysbiosis correlated with the primary and secondary bile acid biosynthesis pathways, implying that it may affect bile acid synthesis in ADT‐su mice, potentially contributing to ADT‐induced cognitive dysfunction. Conversely, gut microbiota in ADT‐uns mice appeared to exert a protective effect against bile acid metabolism disorders. Figure 6F shows the two bile acids (taurodeoxycholic acid [TDCA] and lithocholic acid [LCA]) with significant differences across the groups.

### 3.4 Targeted Bile Acid Metabolomics in the Hippocampus Revealed Bile Acid Metabolic Reprogramming in ADT‐Su Mice

**FIGURE 7:**
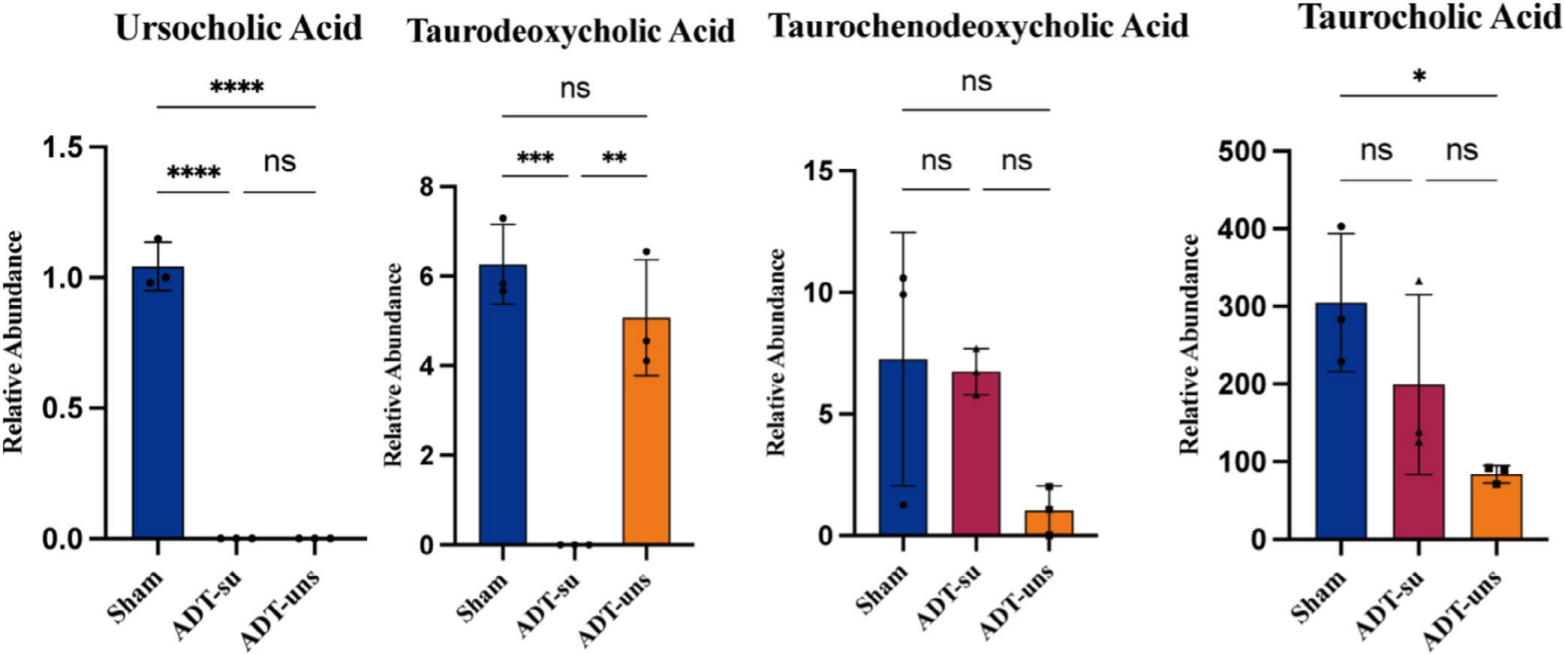
Targeted bile acid metabolomics analysis of the hippocampus in ADT mice. Data are presented as mean ± SEM. Group comparisons of hippocampal bile acid levels were analyzed using one‐way ANOVA with Tukey's multiple comparisons test. *p < 0.05; **p < 0.01; ***p < 0.001; ****p < 0.0001.

Bile acids can normally cross the BBB and infiltrate the central nervous system (CNS), directly or indirectly influencing cognitive‐related processes, including neurotransmitter synthesis and synaptic plasticity. Since the hippocampus is critically involved in cognitive function, we subjected hippocampal bile acids from ADT‐induced cognitive dysfunction mice to targeted metabolomic analysis, revealing significant differences in multiple bile acid levels across the three groups (Figure 7). Notably, compared to the Sham group, ADT induced alterations in the levels of several bile acids within the hippocampus, highlighting its potential impact on bile acid metabolism in mammals. Furthermore, ADT‐su mice exhibited significantly lower TDCA levels in the hippocampus compared to the other two groups, highlighting TDCA's potential protective role against ADT‐induced cognitive dysfunction.

### 3.5 Behavioral Outcomes and Mechanistic Validation After TDCA Supplementation

**FIGURE 8:**
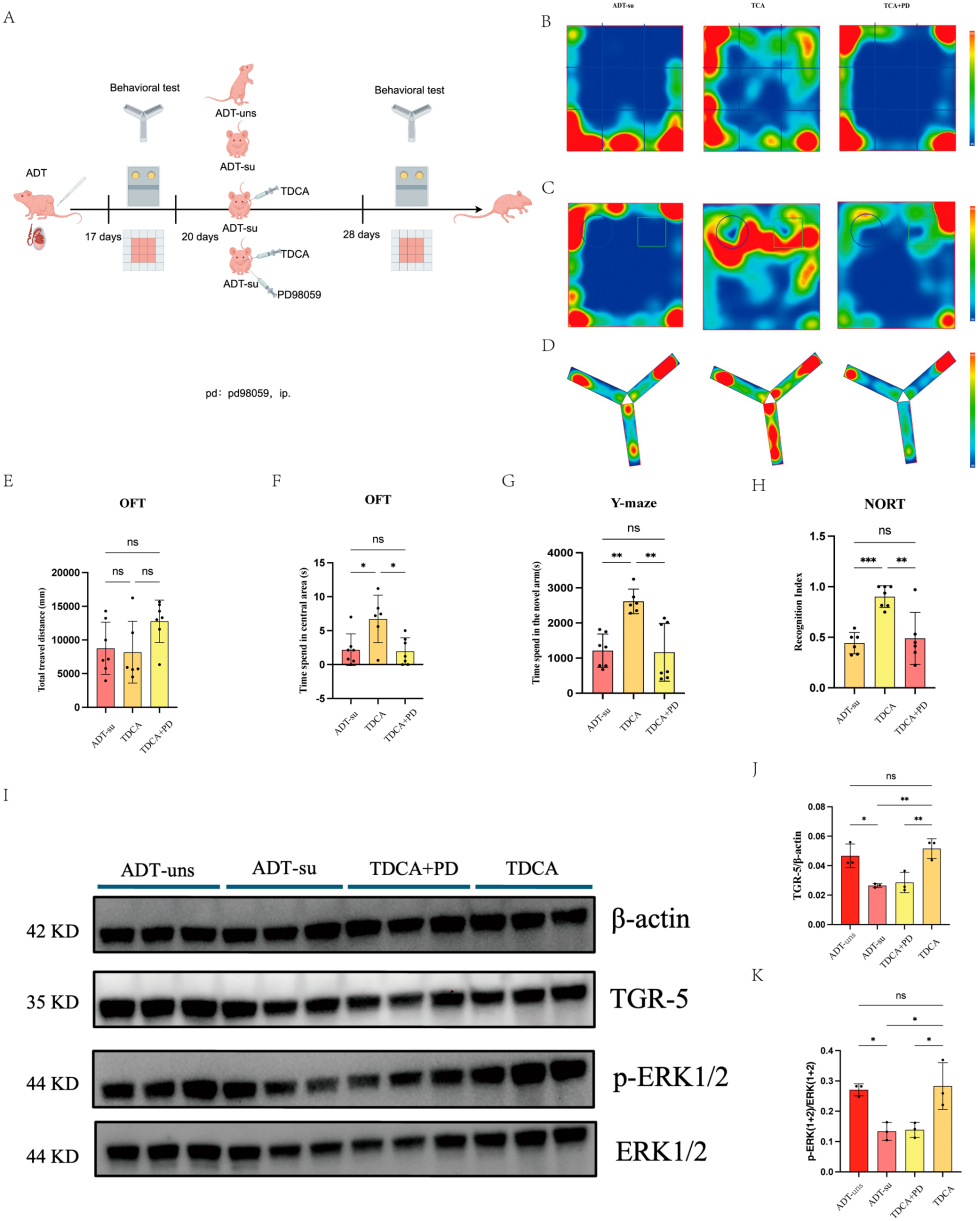
Behavioral and mechanistic validation after TDCA supplementation in the ADT‐induced cognitive dysfunction model. (A) Schematic diagram of the experimental design. Four experimental groups were established: ADT‐uns, ADT‐su, ADT‐su with oral gavage of TDCA, and ADT‐su with TDCA gavage combined with the ERK1/2 inhibitor PD98059 (TDCA + PD98059). (B–D) Representative heatmaps of mouse trajectories during the OFT, NORT, and Y‐maze test. (E, F) Quantification of total distance traveled and time spent in the center area during the OFT. (G) Time spent in the novel arm during the Y‐maze test. (H) Recognition index in the NORT. (I) Representative Western blot images of TGR5, total ERK1/2, phosphorylated ERK1/2 (p‐ERK1/2), and β‐Actin in hippocampal tissues from the four groups. (J, K) Quantification of TGR5 and p‐ERK1/2 expression levels normalized to β‐Actin or total ERK1/2, respectively. Data are presented as mean ± SEM (*n* = 3 per group). *p < 0.05, **p < 0.01; ***p < 0.001; ns, not significant.

To further investigate the involvement of TGR5‐ERK1/2 signaling in the neuroprotective effects of TDCA, we performed Western blot analysis on hippocampal tissues from four experimental groups (ADT‐uns, ADT‐su, ADT‐su + TDCA, and ADT‐su + TDCA + PD98059) (Figure 8A–H). As shown in Figure 8I,J, the expression of TGR5 was significantly elevated in the ADT‐su + TDCA group compared to the ADT‐su group (*p* < 0.01), suggesting enhanced activation of TGR5 by exogenous TDCA supplementation.

Consistently, the level of phosphorylated ERK1/2 (p‐ERK1/2), a key downstream effector of TGR5 signaling, was markedly increased in the ADT‐su + TDCA group relative to ADT‐su controls (*p* < 0.01) (Figure 8I,K). Notably, the administration of PD98059 effectively suppressed TDCA‐induced ERK1/2 phosphorylation (*p* < 0.01 vs. ADT‐su + TDCA), confirming the specificity of ERK1/2 pathway modulation. Correspondingly, the behavioral improvements observed in the ADT‐su + TDCA group were partially reversed in the PD98059‐treated group, further supporting the critical role of the TGR5‐ERK1/2 axis in mediating the cognitive benefits of TDCA.

These findings collectively suggest that the neuroprotective effects of TDCA in ADT‐induced cognitive dysfunction are at least partially dependent on the activation of the TGR5–ERK1/2 signaling pathway.

### 3.6 The Evans Blue Assay Demonstrates BBB Disruption in ADT‐Susceptible Mice

Gross brain inspection revealed minimal or no Evans blue staining in control mice, whereas pronounced blue discoloration was observed in ADT‐treated mice exhibiting cognitive impairment, indicating BBB disruption (Figure S1B). Quantitative measurement confirmed a significant elevation of Evans blue accumulation in ADT‐susceptible mice compared with both control and ADT‐unsusceptible groups (*p* < 0.05, one‐way ANOVA with post hoc test) (Figure S1C). These results demonstrate that ADT‐induced hypogonadism is associated with BBB compromise, which may contribute to the observed neurocognitive deficits.

### 3.7 Serum IL‐6 and TNF‐α Levels Are Selectively Elevated in ADT‐Susceptible Mice

ELISA quantification showed group‐dependent changes in circulating pro‐inflammatory cytokines (Figure S1D). IL‐6 concentrations were markedly increased in ADT‐su mice compared with both control and ADT‐uns groups, whereas ADT‐uns mice displayed a slight but measurable elevation relative to controls. In contrast, TNF‐α levels were significantly higher in ADT‐su mice compared with both control and ADT‐uns groups, while ADT‐uns mice did not differ from controls. These findings indicate that systemic inflammation is most pronounced in ADT‐su mice, aligning with the cognitive impairment phenotype.

## 4 Discussion

It has been established that ADT reduces androgen levels, thus inhibiting PCa, making it a standard intervention, particularly in advanced cases. Besides preventing disease progression, ADT could also improve patient Survival Rates (SRs) [21]. In Castration‐Resistant Prostate Cancer (CRPC) patients, ADT is the first‐line treatment, and its longer duration compared to other PCa cases reflects differences in disease progression. Herein, to align with clinical treatment protocols, we selected the PC3 cell line for studying subcutaneous tumorigenesis. The cell line originated from a bone metastasis of a 62‐year‐old Caucasian male with Stage IV prostate adenocarcinoma, featuring low acid phosphatase activity and 5‐α‐testosterone reductase activity [22]. Unlike androgen‐sensitive cell lines such as LNCaP, PC3 cells do not express androgen receptors (AR), thereby representing the biological features of advanced‐stage prostate cancer. This makes PC3 particularly suitable for investigating the long‐term effects of ADT, especially regarding tumor–host interactions affecting gut microbiota and central nervous system function. Additionally, the subcutaneous implantation approach is technically straightforward, yields a high tumor formation rate, and allows for more consistent monitoring of treatment effects across large sample sizes. After treatment, we assessed the tumor volume in ADT‐treated mice, revealing ADT's minimal impact on tumor growth, which aligns with CRPC characteristics.

Cognitive impairment in PCa patients post‐ADT has recently emerged as a major clinical concern. According to research, ADT could lead to cognitive function decline, with older men experiencing more pronounced impacts [23]. Overall, ADT may affect brain morphology and cognitive function after reducing androgen levels in the body. Although some studies reported no significant changes in working memory or quality of life, long‐term ADT use could lead to more cognitive and neurological consequences [24]. Moreover, research findings on ADT's impact on cognitive function remain somewhat controversial. While some studies have indicated that ADT does not significantly affect patients' cognitive function or reduce depressive symptoms, they still highlight the need for larger and long‐term prospective randomized trials to further validate the findings [25].

To the best of our knowledge, this is the first study to mechanistically link ADT‐induced cognitive dysfunction in PCa mice with gut microbiota dysbiosis and subsequent perturbations in bile acid metabolism. We employed an integrated analytical approach encompassing 16S rRNA sequencing, multi‐compartment metabolomics, and behavioral phenotyping, revealing a novel “gut microbiota‐bile acid‐hippocampal metabolism” axis that drives neurocognitive deficits after androgen deprivation. Notably, our findings in the aforementioned context were consistent with previous studies that linked gut dysbiosis with neurocognitive disorders [26].

We performed hippocampal metabolomic profiling, which revealed significant alterations in bile acid composition, particularly a significant reduction in the levels of neuroprotective bile acids such as TDCA, which demonstrated potential therapeutic effects against neurodegenerative diseases.

Bile acids regulate hippocampal function through two main mechanisms. First, as signaling molecules, they interact with nuclear receptors and GPCRs (e.g., FXR and TGR5) to influence inflammation, neuronal growth, and synaptic plasticity, thereby modulating cognitive function [27], which could mediate their effects on various cellular processes (including inflammation, neuronal growth, and synaptic plasticity), crucially regulating cognitive function. Second, bile acids might function as metabolic modulators of mitochondrial bioenergetics in hippocampal neurons [28]. Mitochondria could crucially impact neuronal functions in various brain regions, including the hippocampus, which regulates learning and memory. Through their effects on mitochondrial function, bile acids might also regulate energy homeostasis in neurons, ensuring adequate energy supply for optimal performance, especially during periods of increased cognitive demand.

16S sequencing revealed that ADT significantly depletes bile acid‐transforming taxa, including Bacteroidetes (e.g., *Bacteroides* spp.) and Firmicutes (e.g., *Clostridium scindens* ). These bacteria are essential for converting primary into secondary bile acids via enzymes such as Bile Salt Hydrolases and 7α‐dehydroxylase [29, 30]. Secondary bile acids, such as LCA and Taurolithocholic Acid (TLCA), are critical modulators of bile acid homeostasis, and their depletion could have far‐reaching effects on metabolic and cognitive functions. Specifically, the depletion of these microbial taxa and their associated enzymatic functions could disrupt the bile acid metabolism balance, compounding the dysregulation of bile acid profiles in the serum and hippocampus. This microbial shift might correlate strongly with altered bile acid profiles in both the serum and hippocampus of ADT‐treated mice, with Spearman correlations ranging from *ρ* = 0.62 to 0.78 and statistical significance (*p* < 0.01). This phenomenon suggests a robust relationship between gut microbiota composition and bile acid metabolism. Furthermore, it mirrors previous clinical observations that linked antibiotic‐induced dysbiosis (microbial imbalance) with an impaired enterohepatic circulation of bile acids [31], a process crucial for maintaining bile acid homeostasis and overall metabolic function. Antibiotic‐induced gut microbiota changes may disrupt bile acid biotransformation, causing primary bile acid accumulation, secondary bile acid reduction, and altered metabolic pathways involved in cognition. Notably, germ‐free mice receiving ADT microbiota (F‐ADT) showed similar bile acid dysregulation and cognitive deficits as donors, while FMT from controls partially restored hippocampal bile acids, suggesting that gut microbiota is a key regulator of systemic and cerebral bile acid homeostasis.

In our study, we observed that levels of TDCA were significantly decreased both in the gut and in the hippocampus of ADT‐susceptible mice, suggesting a potential link between TDCA depletion and cognitive impairment. To further explore this possibility, we reviewed the literature and identified a recent study, which demonstrated that TDCA exerts biological effects via activation of the membrane bile acid receptor TGR5 [32].

Given that TGR5 is expressed in the CNS, including the hippocampus, and modulates pathways such as ERK1/2, we hypothesized that TDCA depletion in ADT‐treated mice may impair TGR5‐mediated neuroprotection. To test this, we supplemented TDCA via oral gavage and selectively inhibited ERK1/2 with PD98059. TDCA supplementation significantly improved cognitive performance in ADT‐susceptible mice, increased hippocampal TGR5 expression, and enhanced ERK1/2 activation, as shown by elevated p‐ERK1/2 levels.

Importantly, administration of PD98059 abolished the TDCA‐induced activation of ERK1/2 and partially attenuated the cognitive improvements, suggesting that the TGR5‐ERK1/2 axis plays a pivotal role in mediating the neuroprotective effects of TDCA in this model. These findings are consistent with prior studies demonstrating that bile acids can modulate central nervous system function through TGR5 activation and downstream MAPK/ERK signaling pathways.

Taken together, our data suggest that targeting the TGR5‐ERK1/2 pathway via bile acid modulation may represent a promising therapeutic strategy to ameliorate cognitive dysfunction associated with ADT treatment in prostate cancer. Future studies are warranted to further elucidate the downstream molecular mechanisms and evaluate the translational potential of TDCA‐based interventions in clinical settings.

Our additional analyses further clarify the role of systemic inflammation and BBB integrity in ADT‐induced cognitive changes. Evans blue extravasation confirmed that BBB permeability is increased in ADT‐susceptible mice, and ELISA results demonstrated significantly elevated circulating IL‐6 and TNF‐α levels in this subgroup. These findings indicate that androgen deprivation can trigger systemic inflammation and compromise BBB integrity, both of which may contribute to neurocognitive deficits.

Importantly, in the FMT experiments, all recipient mice were subjected to identical antibiotic pretreatment, ensuring that FMT was the primary experimental variable. Under these conditions, no significant differences in IL‐6 or TNF‐α levels were observed among recipient groups, suggesting that inflammatory status was effectively controlled and did not confound the cognitive outcomes. This design strengthens our conclusion that gut microbiota alterations, rather than direct systemic inflammatory effects of ADT, underlie the observed behavioral phenotypes.

Despite the comprehensive nature of our study, several limitations should be acknowledged.

First, this work was conducted in a preclinical mouse model. While mouse models are invaluable for elucidating mechanistic insights, inherent interspecies differences in gut microbiota composition, bile acid metabolism, and brain physiology may limit direct translational applicability to human prostate cancer patients receiving ADT. Future validation in clinical cohorts will be essential to confirm these findings in a human context.

Second, we employed a subcutaneous PC3 tumor‐bearing model, which, although technically convenient and reproducible, does not fully recapitulate the complex bone metastatic microenvironment often seen in advanced prostate cancer. The interaction between bone microenvironment, systemic metabolism, and cognitive outcomes warrants further exploration.

Third, while we identified TGR5‐ERK1/2 signaling as a key mediator of the neuroprotective effects of TDCA, the downstream molecular effectors and precise cellular targets within the hippocampus remain to be fully elucidated. Advanced techniques such as single‐cell RNA sequencing and neuronal subpopulation‐specific manipulations could further refine our understanding of these mechanisms.

In summary, our study identifies a novel gut microbiota–bile acid–hippocampus axis underlying ADT‐induced cognitive dysfunction in prostate cancer. We demonstrate that gut microbiota dysbiosis and consequent alterations in hippocampal bile acid metabolism, particularly the depletion of neuroprotective secondary bile acids, contribute to cognitive decline. Furthermore, we provide mechanistic evidence that TGR5‐ERK1/2 pathway activation mediates the beneficial effects of TDCA supplementation on cognition.

These findings not only shed new light on the gut–brain interactions involved in ADT‐associated neurotoxicity but also suggest that targeting bile acid metabolism and TGR5 signaling may represent a promising therapeutic strategy to mitigate cognitive impairment in prostate cancer patients undergoing ADT. Future translational studies are warranted to explore the clinical potential of bile acid‐based interventions and further refine our understanding of this gut–brain–metabolism axis.

## Author Contributions

**Fan Yang:** writing – original draft, methodology, data curation, and conceptualization. **Yanbo Liu:** writing – review and editing and writing – original draft. **Zhien Zhou:** methodology, formal analysis, and data curation. **Dong Yang:** visualization, validation, methodology, formal analysis, and conceptualization. **Weigang Yan:** validation, supervision, and conceptualization.

## Ethics Statement

The animal study was reviewed and approved by Peking Union Medical College Hospital's Animal Care and Use Committee (Approval no: XHDW‐2024‐193).

## Conflicts of Interest

The authors declare no conflicts of interest.

## Supporting information


**Figure S1:** (A) Fecal microbial DNA quantification: antibiotic treatment (ABX, *n* = 18) markedly reduced DNA levels versus controls (CON, *n* = 6; ****p < 0.0001, Mann–Whitney U), confirming gut microbiota depletion. (B) Representative brain images: minimal Evans blue leakage in controls; marked dye penetration in ADT‐susceptible mice, indicating blood–brain barrier disruption. (C) Quantitative Evans blue content (μg/g brain): 3.36 ± 0.9; ****p < 0.0001 versus control, one‐way ANOVA. (D) Cytokine levels: IL‐6 and TNF‐α significantly elevated in ADT‐susceptible mice versus control and ADT‐unsusceptible groups; ADT‐unsusceptible mice show slight or no changes. One‐way ANOVA with Tukey's post hoc (or Kruskal–Wallis with Dunn's correction); *p < 0.05, **p < 0.01, ***p < 0.001, ****p < 0.0001.

## Data Availability

The datasets presented in this study can be found in online repositories. The names of the repository/repositories and accession number(s) can be found below: Untargeted fecal metabolomics: MetaboLights, MTBLS12903 (http://www.ebi.ac.uk/metabolight/MTBLS12903); Targeted serum metabolomics: MetaboLights, MTBLS12904 (http://www.ebi.ac.uk/metabolight/MTBLS12904); Fecal 16S rRNA sequencing: NCBI, BioProject PRJNA1311279 (http://www.ncbi.nlm.nih.gov/bioproject/PRJNA1311279).
