# Supplementary figures and images for: Gut Microbiota–Bile Acid–Brain Axis and TGR5‐ERK1/2 Signaling Mediate ADT‐Induced Cognitive Impairment
Source: CNS Neurosci Ther. 2025 Sep 15;31(9):e70608. doi: 10.1111/cns.70608 (PMC12436682; doi:10.1111/cns.70608)

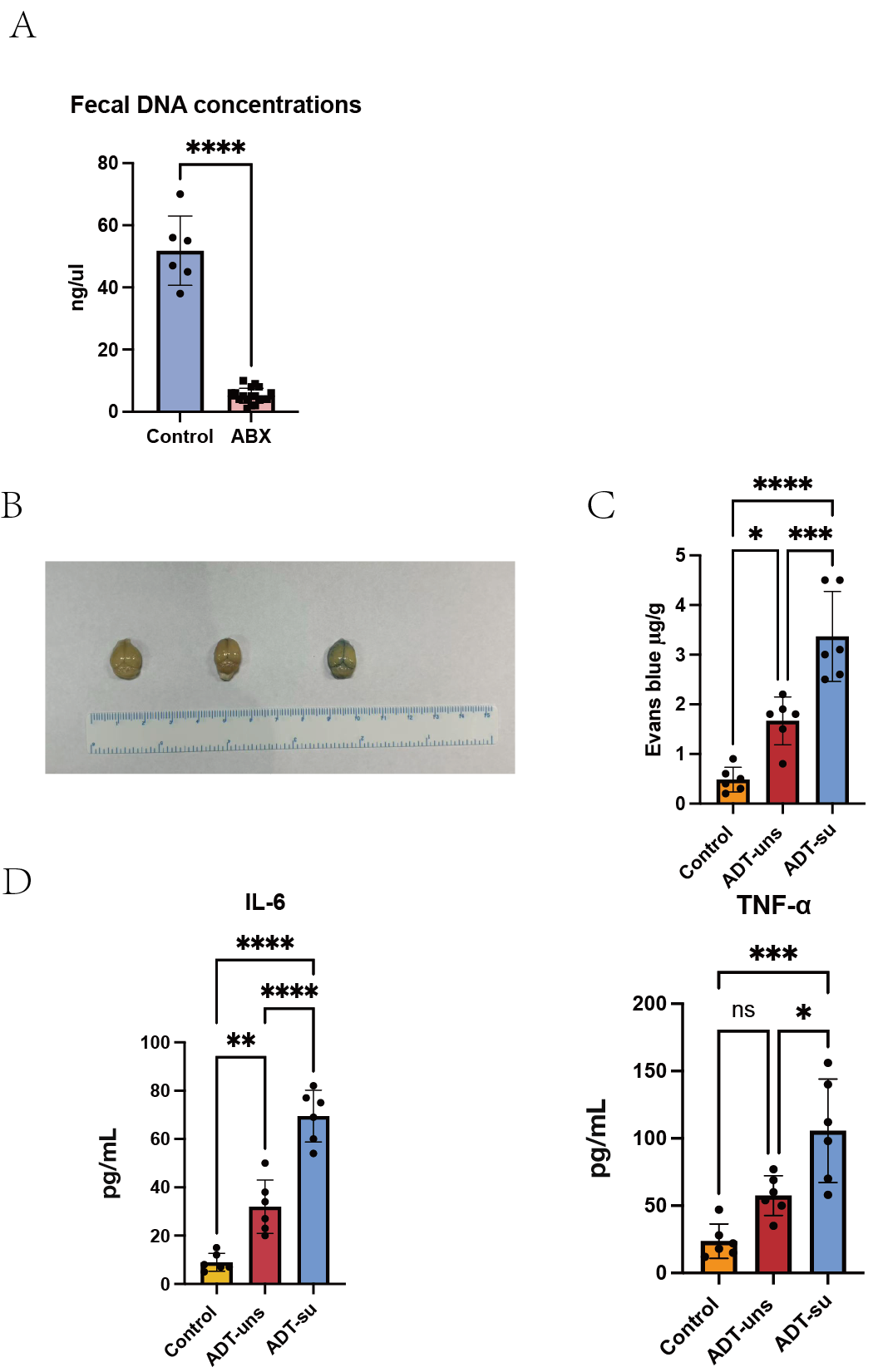

Supplement: Supplementary file 1 — Figure S1: (A) Fecal microbial DNA quantification: antibiotic treatment (ABX, n = 18) markedly reduced DNA levels versus controls (CON, n = 6; ****p < 0.0001, Mann–Whitney U), confirming gut microbiota depletion. (B) Representative brain images: minimal Evans blue leakage in controls; marked dye penetration in ADT‐susceptible mice, indicating blood–brain barrier disruption. (C) Quantitative Evans blue content (μg/g brain): 3.36 ± 0.9; ****p < 0.0001 versus control, one‐way ANOVA. (D) Cytokine levels: IL‐6 and TNF‐α significantly elevated in ADT‐susceptible mice versus control and ADT‐unsusceptible groups; ADT‐unsusceptible mice show slight or no changes. One‐way ANOVA with Tukey's post hoc (or Kruskal–Wallis with Dunn's correction); *p < 0.05, **p < 0.01, ***p < 0.001, ****p < 0.0001. [file CNS-31-e70608-s001.png]
